# Supplementary material for: Oral and Faecal Viromes of New Zealand Calves on Pasture With an Idiopathic Ill-Thrift Syndrome
Source: Transbound Emerg Dis. 2025 Jul 28;2025:7737989. doi: 10.1155/tbed/7737989 (PMC12321419; doi:10.1155/tbed/7737989)
Supplement: Supporting Information 1 — Table SI: Clinical presentations summary of the sampled dairy calves. [file 7737989.f1.docx]

**Supplementary Table 1.** Clinical presentations summary of the sampled dairy calves.

| **Calf VID** | **Oral lesions?** | **Faecal matting or staining?** | **Notes** |
| --- | --- | --- | --- |
| Y7 | N | N |  |
| W3 | N | N |  |
| Y18 | Y | N | “Healthy” but lesion on tongue with diphtheritic |
| Y52 | N | Y | “Healthy” but tail matted |
| Y14 | Y | N | “Healthy” but lesions under tongue |
| W64 | Y | Y | Dental pad lesion? |
| Y41 | Y | N | Clean tail. Lesion under tongue |
| Y17 | Y | Y | Lesion on lower right cheek |
| Y35 | Y | Y | Lesion on top gum |
| Y70 | Y | Y | Lesion on side of cheek, underside of tongue, top of tongue. Diphtheritic material on tongue. |
| P18 | Y | N | Pustules (?) underside of tongue. Ulcers on frenulum. Bovine respiratory syncytial virus? |
