# Supplementary figures and images for: Oral and Faecal Viromes of New Zealand Calves on Pasture With an Idiopathic Ill-Thrift Syndrome
Source: Transbound Emerg Dis. 2025 Jul 28;2025:7737989. doi: 10.1155/tbed/7737989 (PMC12321419; doi:10.1155/tbed/7737989)

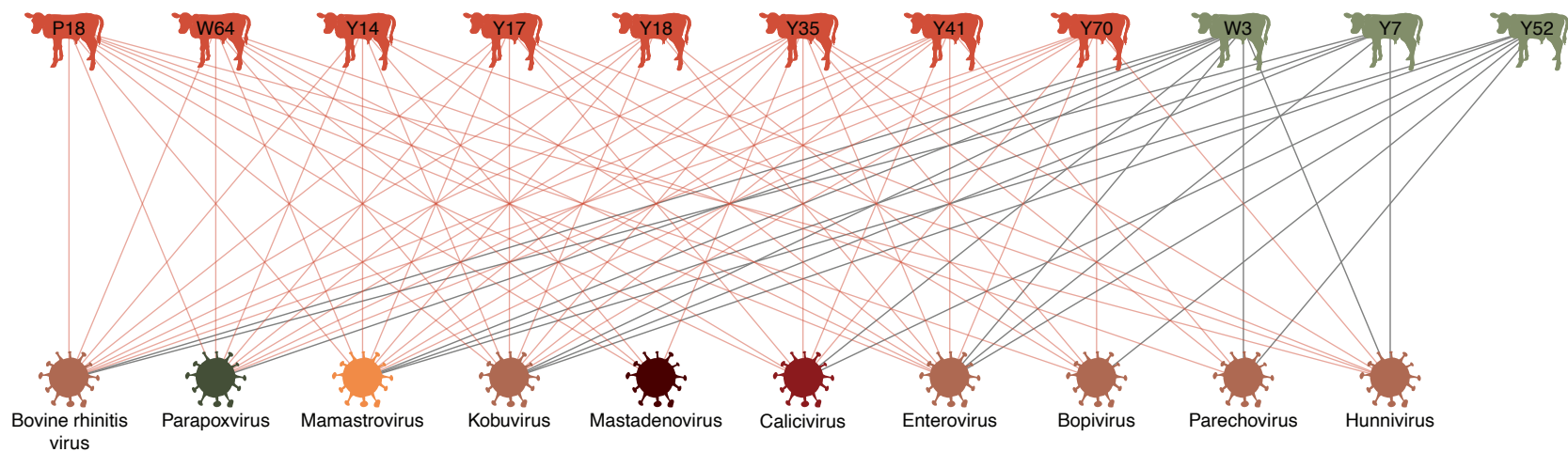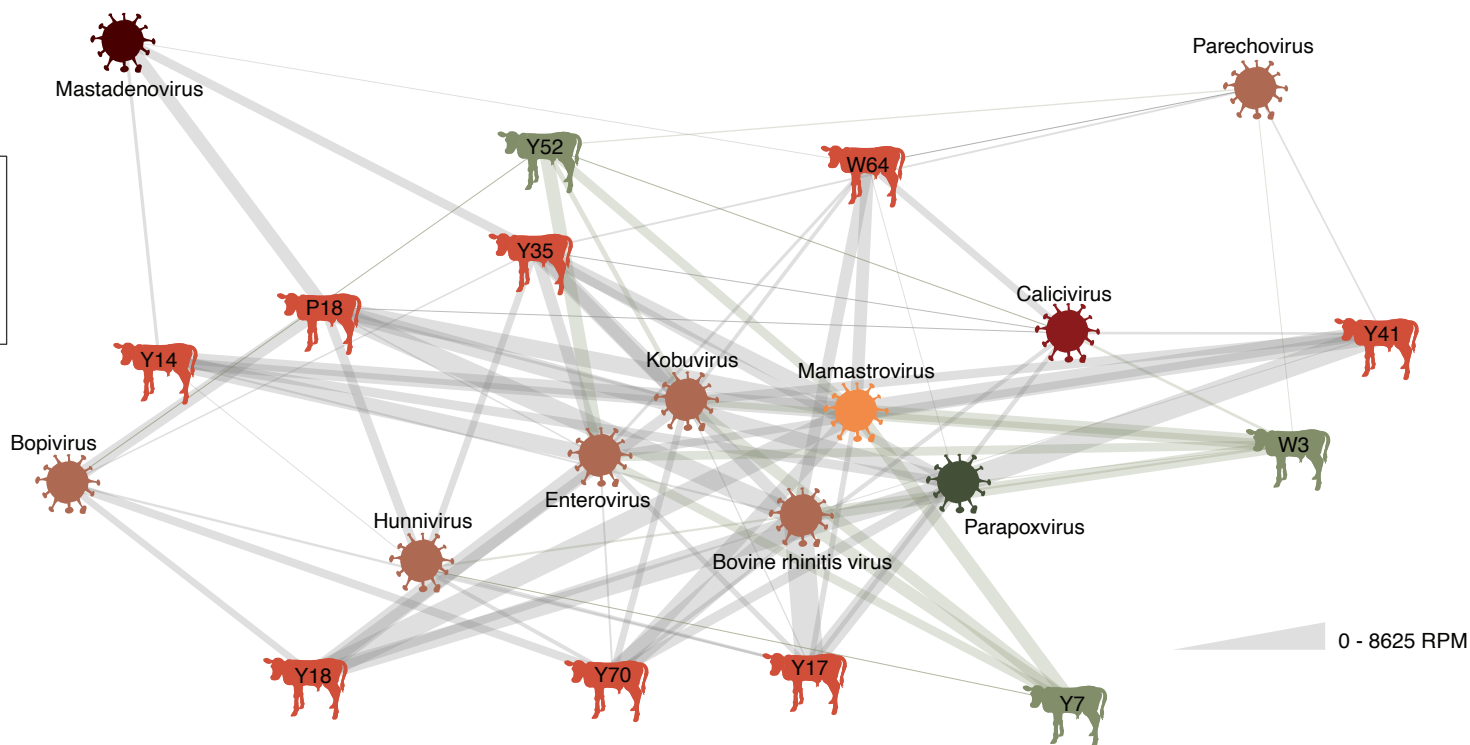

Supplement: Supporting Information 2 — Figure S1: Bipartite networks showing calves and their co-infecting viral genera or species. Railway network showing individual calves and their co-occurring viral groups (top). Weighted network showing co-occurring viruses and their combined abundances in oral and faecal samples in RPM (bottom). Calves unaffected by oral lesions are highlighted in green and calves affected by oral lesions are highlighted in red. [file 7737989.f2.pdf]
